# Relevance of the Carotid Body Chemoreflex in the Progression of Heart Failure

**DOI:** 10.1155/2015/467597

**Published:** 2015-12-08

**Authors:** David C. Andrade, Claudia Lucero, Camilo Toledo, Carlos Madrid, Noah J. Marcus, Harold D. Schultz, Rodrigo Del Rio

**Affiliations:** ^1^Laboratory of Cardiorespiratory Control, Center of Biomedical Research, Universidad Autónoma de Chile, 8900000 Santiago, Chile; ^2^Centro de Fisiología Celular e Integrativa, Clínica Alemana-Universidad del Desarrollo, 7500000 Santiago, Chile; ^3^Department of Physiology and Pharmacology, Des Moines University, Des Moines, IA 50312, USA; ^4^Department of Cellular & Integrative Physiology, University of Nebraska Medical Center, Omaha, NE 68198, USA; ^5^Dirección de Investigación, Universidad Científica del Sur, Lima 15067, Peru

## Abstract

Chronic heart failure (CHF) is a global health problem affecting millions of people. Autonomic dysfunction and disordered breathing patterns are commonly observed in patients with CHF, and both are strongly related to poor prognosis and high mortality risk. Tonic activation of carotid body (CB) chemoreceptors contributes to sympathoexcitation and disordered breathing patterns in experimental models of CHF. Recent studies show that ablation of the CB chemoreceptors improves autonomic function and breathing control in CHF and improves survival. These exciting findings indicate that alterations in CB function are critical to the progression of CHF. Therefore, better understanding of the physiology of the CB chemoreflex in CHF could lead to improvements in current treatments and clinical management of patients with CHF characterized by high chemosensitivity. Accordingly, the main focus of this brief review is to summarize current knowledge of CB chemoreflex function in different experimental models of CHF and to comment on their potential translation to treatment of human CHF.

## 1. Introduction

Chronic heart failure (CHF) is a disease condition characterized by high mortality, frequent hospitalizations, poor quality of life, multiple comorbidities, and complex therapeutic management [1]. Accordingly, CHF is considered a major public health problem throughout the world [2]. In addition, it has been estimated that approximately 20% of the worldwide population will suffer a certain degree of cardiac failure at some point in their lifetime [3].

CHF is characterized by a progressive decrease in cardiac function, which severely impacts blood and oxygen supply to several organs [4–6]. Two pathophysiological hallmarks of CHF are the presence of autonomic imbalance and disordered breathing patterns, both of which are strongly related to the progression of the disease [7–10]. In addition, a heightened carotid body (CB) chemoreflex drive has been shown to play a pivotal role in the development of cardiorespiratory disorders in CHF [11, 12].

Remarkably, it has been shown that CHF patients with an enhanced CB chemoreflex sensitivity have significantly higher mortality rates compared to patients with normal CB chemoreflex sensitivity [12]. In experimental CHF, Del Rio et al. (2013) [13] has shown that elimination of the CB chemoreflex markedly attenuated deterioration of cardiac function and improved survival. Together, these results strongly support a crucial role of the CB chemoreflex in the progression of CHF. The physiological mechanisms related to heightened CB chemoreflex drive in CHF and its deleterious effects are not completely known. Therefore, understanding the contribution of the CB chemoreflex in the pathophysiology of CHF is important to improve current treatments and clinical management of CHF patients and to further develop new therapeutic strategies intended to normalize CB chemoreflex function in CHF. Accordingly, the main focus of this review is to summarize current knowledge of CB chemoreflex function in several CHF models and comment on the potential translational significance to human CHF.

## 2. Carotid Body Chemoreflex and Heart Failure

The CB are the main arterial chemoreceptors involved in cardiovascular and ventilatory adjustments following changes in blood levels of O_2_, CO_2_, pH, and blood flow [14–17]. The CB is organized in clusters of chemoreceptor cells (type I) in charge of sensing bloodstream stimuli, which are surrounded by sustentacular glial cells (type II). The current model of CB chemotransduction theorizes that a chemoreceptor stimulus elicits depolarization of the glomus cells which in turn triggers an increase in [Ca^2+^]_i_ and the release of several neurotransmitters which act on sensory nerve endings projecting centrally from the petrosal ganglion [18]. Chemosensory nerve fibers from the CB project to the nucleus tractus solitarius (NTS), which integrates the CB afferent input [19–21]. Central CB chemoreflex integration takes place in the NTS which in turn sends projections to the respiratory neuronal network and key autonomic nuclei in the brainstem, such as the rostral ventrolateral medulla (RVLM) [22]. In experimental CHF, CB chemoreceptors become tonically active resulting in hyper-activation of RVLM presympathetic neurons and subsequent increases in sympathetic outflow [9, 23].

Importantly, CB chemoreflex activation in CHF is associated with the severity of the disease [12]. Recent studies using selective ablation of the CB chemoreceptors indicate that the CB chemoreflex plays a pivotal role in the cardiorespiratory alterations in experimental CHF [9, 24]. To date, several experimental models of CHF have been used to characterize the molecular and physiological pathways associated with tonic activation of the CB chemoreflex in CHF and its influence on disease progression.

## 3. Experimental Heart Failure Models

There are numerous experimental models of CHF that recapitulate many of the pathophysiological abnormalities that occur in human CHF (Table 1). While murine models are the most widely used, rabbits, sheep, and dogs have also been used to study CHF. In the paragraphs to follow we review what is known about the role of CB chemoreflex function in autonomic and respiratory alterations. Also, we discuss the potential mechanisms related to the development of heightened CB chemosensory function in CHF.

### 3.1. Myocardial Infarction Model

In the myocardial infarction-induced CHF model (MI-CHF), heart failure is generated through the surgical induction of ischemia in cardiac tissue. Two experimental approaches have been used. The first approach is characterized by electrocauterization of the epicardial surface to induce small focal infarctions [25]. The second and more frequently used experimental approach requires ligation of the descending coronary artery [26]. It has been shown that MI-CHF rats display an increase CB chemoreflex and CB chemoreceptor activity within 6–8 weeks of infarction [9, 27]. In addition, MI-CHF rats develop autonomic imbalance characterized by changes in heart rate variability, increased renal sympathetic nerve activity, and increases in circulating norepinephrine levels (Table 2) [28–30]. Moreover, an increased incidence of respiratory disorders is also observed in MI-CHF rats (Table 3) [31]. Importantly, Del Rio et al. (2013) [13] showed for the first time that selective bilateral CB denervation in MI-CHF rats decreased the activity of presympathetic neurons of the RVLM, reversed autonomic imbalance, and markedly reduced mortality risk. Taken together, these findings indicate that CB chemoreflex plays an important role in the pathophysiology of the MI-CHF model.

### 3.2. Rapid Ventricular Pacing Model

The rapid-pacing CHF (RP-CHF) model is characterized by a tachycardia-induced cardiomyopathy. This CHF model produces elevated ventricular filling pressures and reduced systolic and diastolic ventricular function. Additionally, this model is associated with intense neurohumoral activation and disordered breathing patterns (Shinbane et al. 1997) [32]. Sun et al. (1999) [23] showed that 3 weeks of rapid pacing was necessary to induce CHF in rabbits. Li et al. (2005) [33] showed that RP-CHF rabbits displayed enhanced CB chemoreflex function evidenced by increases in both sympathetic nerve activity [33] and ventilatory responses to acute hypoxic stimulation [34]. Additionally, cardiac autonomic imbalance was also shown in this model by means of reductions in the total power of heart rate variability (Table 2) [35]. Recently, Marcus et al. (2014) [24] provided compelling evidence that the CB chemoreceptors play a pivotal role in the progression of RP-CHF. In this model, CB denervation performed after the development of CHF significantly reduced renal sympathetic nerve activity and incidence of disordered breathing patterns, restored cardiac autonomic balance, and reduced exaggerated respiratory-sympathetic coupling (Table 3) [10, 24].

### 3.3. Ascending Aortic Constriction Model

Banding of the ascending aorta is an experimental technique to produce a pressure-overload form of CHF (AB-CHF). This surgical approach requires reducing aortic diameter by tying a suture around the ascending aorta [36]. Banded animals develop hypertension and left ventricular hypertrophy. After 18 weeks, the banded animals have clear signs of CHF [37]. The CB chemoreflex has not been studied in AB-CHF animals; however it has been shown that hypoxic stimulation induced an increase in the left ventricular end diastolic pressure [38]. This result suggests that CB activation may play a role in the regulation of cardiac function in AB-CHF. In addition, results showing that AB-CHF rats displayed an increased renal sympathetic nerve activity in response to hypercapnic stimulation suggest a plausible contribution of central and/or CB chemoreflex pathways in the regulation of sympathetic outflow [39]. Further studies are needed to determine if the CB chemoreflex pathway plays any role in the progression of AB-CHF.

### 3.4. Dilated Cardiomyopathy Genetic Model

Genetic models of CHF are less common; however one genetic CHF model (G-CHF) expresses a dominant-negative form of the basic leucine zipper CREB transcription factor CREB_A133_ (Ser-Ala^133^) [40]. Mutant mice showed clear signs of CHF with the presence of cardiac hypertrophy and neurohumoral activation. Importantly, G-CHF mice showed an increased CB chemoreceptor activity and chemoreflex response to hypoxia [41]. Additionally, breathing regularity was markedly impaired compared to the ventilatory rhythm observed in normal mice (Table 3). Also, G-CHF mice displayed ventricular arrhythmias that were normalized by denervation of the CB chemoreceptors [41]. This result strongly suggests that the CB chemoreflex contributes to the development of cardiac arrhythmias.

### 3.5. Aorto-Caval Shunt Model

Volume overload is commonly used to induce CHF with preserved ejection fraction [42]. The most used animal model is the aorto-caval shunt CHF model (ACS-CHF). Here an arteriovenous shunt is surgically created between the inferior vena cava and the abdominal aorta to induce a significant cardiac volume overload [43]. This experimental approach leads to diastolic CHF and is characterized by neurohumoral activation and sympathetic hyperactivity (Table 2) [44]. The contribution of the CB chemoreflex in the development of cardiorespiratory impairment in ACS-CHF has not previously been studied. Kristen et al. (2002) [39] showed that hypercapnic stimulation triggered a modest sympathetic response in rats with ACS-CHF. This result suggests that central and/or CB chemoreceptors may regulate autonomic balance in ACS-CHF. To date, breathing instability has not been evaluated in this model (Table 3). Future studies should focus on the understanding of the contribution of the CB and central chemoreceptors in the progression of autonomic imbalance in ACS-CHF.

## 4. Mechanisms of Altered Carotid Body Function in CHF

While the mechanisms underpinning CB potentiation in CHF are not fully understood it has been widely accepted that angiotensin peptides and oxidative stress both play a major role in the enhanced CB chemoreflex drive observed in CHF (for review see [45–48]). Circulating angiotensin II (AngII) levels are significantly higher during the progression of CHF. In addition, the presence of a local angiotensin production system in the CB has been described [47] and could contribute as well. In support of this notion, AngII levels are higher in the CBs from CHF rabbits compared to controls [33]. It has been proposed that AngII could alter CB function in CHF by altering redox balance, as increased circulating or local AngII could increase production of superoxide (O_2_^•−^) radical via activation of the AT1_R_ [33]. Indeed, it has been shown that AT1_R_ blockers effectively reduced CB afferent activity in CHF [33]. The mechanisms that subsided the effects of AngII on CB function have been related to NADPH oxidase-dependent O_2_^•−^ production since application of phenylarsine oxide (an NADPH oxidase inhibitor) significantly reduced CB chemosensory afferent activity [49]. Furthermore, the molecular mechanism that relates AngII with changes in CB chemoreceptor cell excitability has also been described [50]. In CHF, increases in AngII-dependent oxidative stress inhibit voltage gated K^+^ channels and depolarize CB glomus cells [50]. In addition to increases in prooxidant factors, during CHF the CBs also undergo a marked reduction in the expression of antioxidant enzymes. Indeed, CuZn- and Mn-SOD enzymes, two important cellular scavenger of O_2_^•−^ [51], have been shown to be downregulated in the CB from CHF rabbits [52]. Accordingly,* in vivo* CB transfection with CuZn- and Mn-SOD transgenes restores normal CB chemoreceptor cells excitability by normalizing resting membrane potential to values comparable to the ones obtained in control CBs [53]. Taken together, these findings show that AngII and oxidative stress contribute to altered CB function in CHF.

In addition to AngII, endothelin 1 (ET-1), another potent vasoactive peptide, has been shown to be constitutively expressed within the CB tissue along with its type A (ET-A_R_) and B (ET-A_R_) receptor [54–56]. Furthermore, ET-1 mediated signaling through the ET-A_R_ has been shown to enhance the CB afferent activity [54]. Moreover, in intermittent hypoxia mimicking obstructive sleep apnea (OSA) model, ET-1 and ET-A_R_ have been shown to mediate CB chemosensory potentiation [54, 55]. Interestingly, OSA and CHF are both characterized by the presence of an enhanced CB afferent activity and autonomic imbalance [27, 57]. Despite this evidence, the contribution of ET-1 and endothelin receptors in CHF has not been studied. However, ET-1 levels have been found to be increased in the plasma of CHF patients [58]. Therefore, it is plausible that increased ET-1 levels in experimental CHF could also play a role in enhancing CB chemosensory afferent activity. Further studies are needed to uncover the role of ET-1 on CB chemosensory function in CHF.

Recently, a CB type II cell-dependent modulation of glomus cell function has also been described [59, 60]. This novel mechanism seems to be related to the activation of the type II cell and the further paracrine secretion of the putative neurotransmitter ATP to the vicinity of glomus cells and sensory nerve endings [60]. Interestingly, type II cells as well as glomus cells display AT1_R_ expression [59]. Then, it is plausible that local and/or systemic increases in AngII levels during the progression of CHF could activate type II cells causing ATP release and chemosensory excitation. Future studies should focus on the role of CB type II cells in the augmented CB chemosensory afferent activity during CHF.

## 5. Conclusions and Perspective

CHF is characterized by sympathetic hyperactivity independent of the etiology of the cardiac failure. In addition, it has been shown that a significant proportion of CHF patients displays elevated CB chemoreflex drive [12]. Several CHF experimental models also display heightened CB chemoreflex drive, and this is positively correlated with the severity of the disease. Recent exciting studies indicate that ablation of the CB chemoreceptors not only improves autonomic function and reduces disordered breathing patterns in experimental CHF but also improves survival. More importantly, Niewiński et al. (2013) [53] has recently shown the relevance of the CBs in human CHF. In a pilot study with one CHF patient (NYHA class II) they show that CB denervation is an effective therapeutic strategy to reduce the progression of the disease. Two and six months after CB denervation the patient showed clear signs of an improvement in autonomic control (total heart rate variability and baroreflex gain), sleep breathing disorders (apnea/hypopnea score), exercise tolerance, and an important improvement in his quality of life [51]. Together, preclinical and clinical studies unveil the relevance of the CB chemoreflex in the progression of systolic CHF. These findings raise the question of whether the CB chemoreflex should be tested in all forms of CHF (i.e., systolic versus diastolic). Unfortunately, CB chemoreflex function has not been investigated in experimental models of diastolic CHF. Taking into account the impressive results of previous studies showing the benefits of CB denervation in experimental and human systolic CHF, future studies addressing the role of the CB in the progression of autonomic imbalance and disordered breathing patterns in nonsystolic CHF are important for the development of future strategies intended to improve quality of life and survival in these patient populations.

## Figures and Tables

**Table 1:** Incidence of autonomic imbalance, breathing disorders and carotid body chemoreflex potentiation in experimental CHF.

	Autonomic imbalance	Breathing disorders	Altered CB chemoreflex	References
MI-CHF	•	•	•	[13]
RP-CHF	•	•	•	[35]
AB-CHF	•	—	—	[39]
G-CHF	•	•	•	[41]
ACS-CHF	•	—	—	[44]

•: described in the literature; —: not described in the literature. MI-CHF: myocardial infarct chronic heart failure; RP-CHF: rapid pacing chronic heart failure; AB-CHF: aortic banding chronic heart failure; G-CHF: genetic chronic heart failure; ACS-CHF: aortocaval shunt chronic heart failure.

**Table 2:** Hemodynamic, autonomic balance, and baroreflex function in CHF models.

	Hemodynamic		Autonomic balance			Baroreflex		References
	BP	HR	U-NE	HRV	Blockers	Oxford	BRS	
MI-CHF	—	—	↑	↓	Symp. ↑ Parasymp. ↓	↓	↓	[13, 55, 61]
RP-CHF	↓	↑	↑	↓	Symp. ↑ Parasymp. ↓	—	↓	[33–35, 61, 62]
AB-CHF	↑	↑	↑	ND	—	ND	—	[39]
G-CHF	ND	—	ND	↓	ND	ND	ND	[41]
ACS-CHF	↓	—	↑	ND	ND	↓	ND	[39, 42]

BP: blood pressure; HR: heart rate; HRV: heart rate variability; U-NE: urinary norepinephrine; Blockers: Propranolol/Atropine test; Oxford: baroreflex test address by phenylephrine and sodium nitroprusside i.v. infusion; BRS: spontaneous baroreflex sensitivity; ND: not described; ↑: increased; ↓: decreased; and —: without difference compared to control healthy condition. MI-CHF: myocardial infarct chronic heart failure; RP-CHF: rapid pacing chronic heart failure; AB-CHF: aortic banding chronic heart failure; G-CHF: genetic chronic heart failure; ACS-CHF: aortocaval shunt chronic heart failure.

**Table 3:** Periodic breathing, breathing irregularity, and apnea/hypopnea score in experimental CHF.

	Periodic breathing	Breathing irregularities	Apnea/hypopnea index	References
MI-CHF	ND	↑	↑	[26]
RP-CHF	↑	↑	↑	[35]
AB-CHF	ND	ND	ND	
G-CHF	ND	↑	↑	[41]
ACS-CHF	ND	ND	ND	

ND: not described; ↑: increased; ↓: decreased; and —: without difference compared to control healthy condition. MI-CHF: myocardial infarct chronic heart failure; RP-CHF: rapid pacing chronic heart failure; AB-CHF: aortic banding chronic heart failure; G-CHF: genetic chronic heart failure; ACS-CHF: aortocaval shunt chronic heart failure.
